# Hspa13 Deficiency Impaired Marginal Zone B Cells Regulatory Function and Contributed to Lupus Pathogenesis

**DOI:** 10.1002/advs.202413144

**Published:** 2024-12-31

**Authors:** Chen Xing, Haoran Cui, Ge Li, Xiaoling Liu, Kun Liu, Qing Wen, Xin Huang, Renxi Wang, Lun Song

**Affiliations:** ^1^ Beijing Institute of Basic Medical Sciences Beijing 100850 China; ^2^ Department of Dermatology First Medical Centre of Chinese PLA General Hospital Beijing 100853 China; ^3^ Beijing Institute of Brain Disorders, Laboratory of Brain Disorders, Ministry of Science and Technology Collaborative Innovation Center for Brain Disorders Capital Medical University Beijing 100069 China

**Keywords:** Hspa13, IL‐10, MZ B cells, regulatory B cells, regulatory T cells, SLE

## Abstract

Dysregulated IL‐10 producing regulatory B cells (Bregs) are associated with the progression of systemic lupus erythematosus. An immunomodulatory role of heat shock proteins (HSPs) is implicated in autoimmune diseases. However, the molecular basis underlying the role of Hspa13 in regulating Bregs function and lupus pathogenesis remains unclear. In this study, Bregs display higher Hspa13 expression than IL‐10^−^ B cells. Induction of IL‐10 production is weakened in B cells with Hspa13 knockdown or knockout. Hspa13 binds to the IL‐10 promoter via the TATA or CAAT box and activates IL‐10 transcription in the nucleus. Furthermore, Hspa13 positive cells are enriched in marginal zone (MZ) B cells to regulate IL‐10 production. Stimulated B220^+^ B or MZ B cells from CD19^cre^Hspa13^fl/fl^ mice for Breg induction show an impaired capacity to promote CD4^+^Foxp3^+^ regulatory T cells (Treg) differentiation. In lupus MRL/lpr mice, a decline in Treg differentiation is accompanied by decreased Hspa13 expression in both Bregs and MZ B cells. Moreover, adoptive transfusion of Bregs and MZ B cells from CD19^cre^Hspa13^fl/fl^ mice fails to increase the frequency of Tregs, attenuate renal pathology, or decrease anti‐dsDNA antibody levels. These results explain the unique role of Hspa13 in determining MZ regulatory function and affecting lupus pathogenesis.

## Graphical abstract

Hspa13 can be induced to regulate IL‐10 production in MZ B cells and support Tregs differentiation. Functional decline of Bregs is accompanied with decreased Hspa13 expression in Bregs and MZ B cells in lupus MRL/lpr mice. Hspa13 deficiency in B cells derived from the CD19^cre^Hspa13^fl/fl^ mice impaired their regulatory functions and therapeutic roles in improving the symptoms of lupus mice.

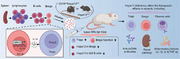

## 1 Introduction

Systemic lupus erythematosus (SLE) is a complex chronic autoimmune disease characterized by excessive autoantibody production and heterogeneous clinical manifestations with multiorgan involvement.^[^ ^1^ ^]^ Abnormal B cell function in autoantibody production plays a key role in the pathogenesis of SLE. Besides, abnormal regulatory B cells (Bregs) have been identified during the development of SLE.^[^ ^2^ ^]^ Bregs exhibit immune suppressive functions by releasing anti‐inflammatory cytokines such as IL‐10, IL‐35, or transforming growth factor (TGF)‐β, directly interacting with pro‐inflammatory immune cells, or inducing regulatory T cells (Tregs).^[^ ^3^, ^4^ ^]^ The IL‐10‐producing regulatory B cells are the most widely studied Bregs subsets, whose phenotypes have been identified in CD1d^hi^CD23^−^IgM^hi^CD1d^hi^ marginal zone (MZ) B cells,^[^ ^5^, ^6^ ^]^ CD1d^hi^CD23^+^IgM^hi^CD1d^hi^ T2 marginal zone precursor (T2‐MZP) B cells,^[^ ^7^ ^]^ TIM‐1^+^ B cells,^[^ ^8^ ^]^ plasmablasts,^[^ ^9^ ^]^ plasma cells,^[^ ^10^ ^]^ and CD5^+^ B‐1a B cells in mice,^[^ ^11^ ^]^ and CD24^hi^CD27^+^,^[^ ^12^ ^]^ and CD24^hi^CD38^hi^ B cells in human.^[^ ^13^ ^]^ However, findings on Bregs in SLE pathogenesis are complicated due to the differences in Breg phenotypes and heterogeneity in patients with lupus. For example, some studies have found increased circulating Breg numbers and serum IL‐10 levels in patients with SLE.^[^ ^14^, ^15^ ^]^ While, other studies have also described decreased numbers or impaired functions of Breg subsets in patients with SLE.^[^ ^13^, ^16^ ^]^ Thus, further elucidation on the critical factors regulating IL‐10 production and functional features of Bregs are still necessary to understand SLE pathogenesis.

Heat shock proteins (HSPs) are highly conserved proteins with high homology from prokaryotes to higher eukaryotes. Most HSPs are highly stress‐inducible and play cytoprotective roles upon exposure to various stressful conditions. The classical roles of HSPs are they function as intracellular chaperones in maintaining proteostasis.^[^ ^17^ ^]^ Notably, HSPs can act as immunogenic proteins and are involved in the pathogenesis of autoimmune diseases, including SLE.^[^ ^18^, ^19^, ^20^ ^]^ The immunomodulatory effects of HSPs, which regulate cytokine production or induction of IL‐10 producing T cells or CD4^+^Foxp3^+^Tregs, have been explored in autoimmune and some immune disorders.^[^ ^21^ ^]^ However, the immunoregulatory function of HSPs in B cells remains unclear.

Hspa13, also known as the stress chaperone (STCH), is a member of the HSP70 family. Studies have suggested that Hspa13 acts as a scaffolding factor to regulate the endoplasmic reticulum and cytosolic proteostasis by modulating protein translocation and stability.^[^ ^22^, ^23^, ^24^ ^]^ One study revealed that Hspa13 exhibited antiviral effects by promoting production of IFN‐β and interferon‐stimulated genes in macrophages.^[^ ^25^ ^]^ Our previous findings have verified the involvement of Hspa13 in lupus‐prone mice and its role in the regulation of plasma cell production and antibody secretion.^[^ ^26^ ^]^ However, current knowledge regarding the biological function of Hspa13 in regulating immune responses remains limited. In particular, the contribution of Hspa13 to the disruption of immune tolerance in SLE, and whether Hspa13 plays a role in the immune regulatory function by regulating IL‐10‐producing Breg function remains unclear.

To examine the potential role of Hspa13 in regulating IL‐10‐producing Breg function and lupus disease progression in MRL/lpr mice, we used a previously established CD19^cre^Hspa13^fl/fl^ mouse line with Hspa13 deletion in B cells.^[^ ^26^ ^]^ We identified Hspa13 as a novel positive regulator of IL‐10‐producing Bregs and found that it was mostly enriched in the marginal zone (MZ) B cells. Hspa13 deficiency in MZ B cells contributed to lupus pathology by reducing IL‐10 production and the proportion of Tregs. Thus, our results illustrated the vital role of Hspa13 in promoting IL‐10‐producing Breg induction and its regulatory function, and partially elucidated the reason for the impaired regulatory function of MZ B cells in lupus‐prone mice.

## 2 Results

### 2.1 IL‐10‐Producing B Cells Displayed High Hspa13 Expression

**Figure 1:**
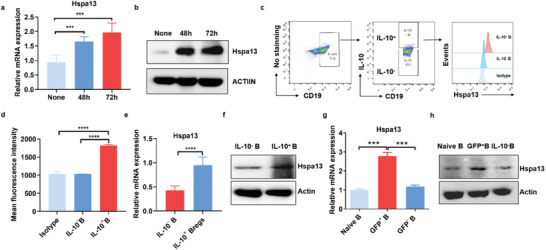
IL‐10‐producing B cells displayed high Hspa13 expression. a) The induction of IL‐10‐producing B cells in vitro was conducted via stimulation of isolated B cells using LPS (10 µg mL^−1^) and cell activation cocktail (PMA (50 ng mL^−1^), Ionomycin (1 µg mL^−1^). With the induction of IL‐10^+^ Bregs, Hspa13 mRNA expression in stimulated B cells greatly increased by real‐time PCR analysis (*n* = 6, ^***^ *p *< 0.001). b) Protein expression of Hspa13 elevated in stimulated B cells as observed by western blot analysis. c) IL‐10 positive B cells displayed a higher Hspa13 level compared to the IL‐10 negative B cells using indirect immunofluorescence staining through FACS. d) The mean fluorescence intensity of Hspa13 abundance in IL‐10^+^ B cells was significantly higher than that in IL‐10^−^B cells via the FlowJo X software analysis (*n* = 3, ^****^ *p *< 0.0001). e) IL‐10^+^ Bregs were induced, and then purified using a Bregs isolation kit. Hspa13 mRNA expression exhibited a significant increase in IL‐10^+^ Bregs compared to the IL‐10^−^ B cells using real‐time PCR analysis (*n* = 6, ^****^ *p *< 0.0001). f) Hspa13 protein expression was much higher in IL‐10^+^ Bregs compared to that in the IL‐10^−^ B cells observed through western blot analysis. g,h) GFP^+^ B cells directly indicating IL‐10^+^ Bregs were isolated from IL‐10‐EGFP reporter tiger mice through FACS analysis. Hspa13 expression in GFP^+^ B cells was much higher than that in naive B cells or GFP^−^ B cells (*n* = 3, ^***^ *p *< 0.001). Statistical analyses were performed by two‐tailed *t*‐tests between two groups, unless otherwise stated.

To explore the potential function of Hspa13 in regulating IL‐10 production in B cells, we first analyzed the expression of Hspa13 in IL‐10‐producing B cells. The induction of IL‐10‐producing B cells in vitro was conducted via stimulation of isolated B cells with LPS and a cell activation cocktail, as previously described.^[^ ^27^, ^28^ ^]^ With the induction of IL‐10^+^ Bregs in vitro, Hspa13 mRNA and protein expression both increased significantly (**Figure** **1**a,b, Figure S1a, Supporting Information). To better understand the Hspa13 expression pattern in B cells, we conducted indirect immunofluorescence staining to analyze Hspa13 abundance in IL‐10‐producing B cells and IL‐10 negative B cells separately by fluorescence‐activated cell sorting (FACS). Within CD19^+^ B cells, Hspa13 expression was further analyzed in gated IL‐10 positive B cells and IL‐10 negative B cells (Figure 1c). The mean fluorescence intensity indicated that Hspa13 abundance in IL‐10 positive B cells was significantly higher than that in IL‐10 negative B cells (Figure 1c,d). We then analyzed Hspa13 expression in IL‐10^+^ Bregs and IL‐10^−^ B cells isolated from stimulated B cells using the Breg isolation kit. The results showed that Hspa13 mRNA and protein expression significantly increased in IL‐10^+^ Bregs compared to that in the IL‐10^−^ B cells (Figure 1e,f; Figure S1b, Supporting Information). Additionally, we also utilized IL‐10‐EGFP reporter tiger (interleukin‐ten ires gfp‐enhanced reporter) mice and isolated GFP^+^ B cells to directly indicate IL‐10^+^ Bregs. Similarly, GFP^+^ B cells showed higher Hspa13 expression than that in naive B cells or GFP^−^ B cells (Figure 1g,h; Figure S1c, Supporting Information). Thus, IL‐10‐producing B cells display much higher Hspa13 expression compared to the IL‐10^−^ B cells.

### 2.2 Hspa13 Regulated IL‐10 Production in B Cells

**Figure 2:**
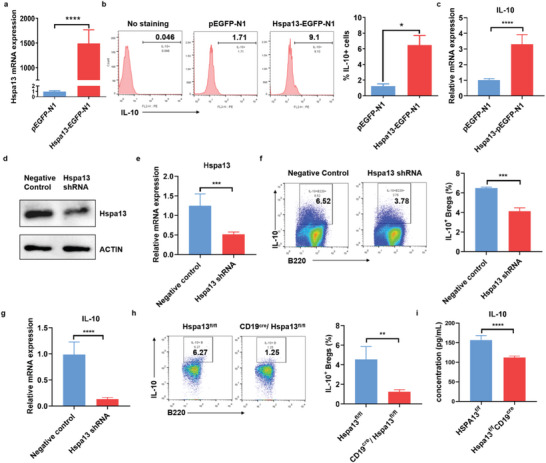
Hspa13 regulated IL‐10 production in B cells. a) Hspa13 was overexpressed in CHO cells via Hspa13‐pEGFP‐N1 vector transfection, and Hspa13 expression was detected by real‐time PCR analysis (*n* = 6, ^****^ *p *< 0.0001). b) The proportion of IL‐10^+^ cells significantly increased in Hspa13 overexpressed cells compared to the control group by FACS analysis (^*^ *p *< 0.05). c) IL‐10 transcriptional expression was higher in the Hspa13‐pEGFP‐N1 vector transfected cells than that in the pEGFP‐N1 vector transfected cells (*n* = 6, ^*^ *p *< 0.0001). d) Hspa13 protein level and e) mRNA expression were knocked down in B cells via Hspa13‐specific shRNA‐expressing lentiviral infection (*n* = 6, ^*^ *p *< 0.001). f) Hspa13 knockdown induced the decreased IL‐10^+^ B cells proportion as observed by FACS analysis (*n* = 3, ^*^ *p *< 0.001). g) Hspa13 knockdown reduced IL‐10 mRNA expression in B cells (*n* = 6, ^*^ *p *< 0.0001). h) B cells with genetic Hspa13 deficiency isolated from spleens of the CD19^cre^Hspa13^fl/fl^ mice greatly impaired their capacity in induction of IL‐10^+^ B cells compared to that from the Hspa13^fl/fl^ mice (*n* = 4, ^*^ *p *< 0.01). i) B cells were stimulated for the induction of IL‐10 production. IL‐10 concentration in the culture medium of stimulated B cells from CD19^cre^Hspa13^fl/fl^ mice decreased compared to that in the control (*n* = 6, ^*^ *p *< 0.0001). Statistical analyses were performed by two‐tailed t‐tests between two groups, unless otherwise stated.

To explore the regulation of Hspa13 on IL‐10 production, we overexpressed Hspa13 in CHO cells (**Figure** **2**a). The proportion of IL‐10^+^ cells and IL‐10 transcriptional expression significantly increased in Hspa13 overexpressed cells compared to those in the control group (Figure 2b,c). To further verify the regulation of Hspa13 on IL‐10 production in B cells, isolated B220^+^ B cells with Hspa13 expression knockdown were stimulated to induce IL‐10 production. Western blot and q‐PCR analyses showed that Hspa13 expression dramatically decreased in B cells through Hspa13‐specific shRNA‐expressing lentiviral infection (Figure 2d,e; Figure S1d, Supporting Information). As expected, Hspa13 knockdown decreased the proportion of IL‐10^+^ B cells as determined by FACS analysis (Figure 2f). Meanwhile, qPCR analysis revealed that IL‐10 mRNA levels significantly reduced following Hspa13 knockdown (Figure 2g). To further examine the potential role of Hspa13 in regulating IL‐10‐producing Breg function, we used the previously established CD19^cre^Hspa13^fl/fl^ mouse line with Hspa13 deletion in B cells.^[^ ^26^ ^]^ Hspa13 deficiency in B cells isolated from the spleens of CD19^cre^Hspa13^fl/fl^ mice also greatly impaired the induction of IL‐10^+^ B cells compared to that in the Hspa13^fl/fl^ mice (Figure 2h). The concentration of IL‐10 in the culture medium of stimulated B cells from CD19^cre^Hspa13^fl/fl^ mice was lower than that in the control (Figure 2i). Collectively, these results indicated that Hspa13 is a positive regulator of IL‐10 production in B cells.

### 2.3 Hspa13 Could Bind to IL‐10 Promoter to Activate IL‐10 Transcription

**Figure 3:**
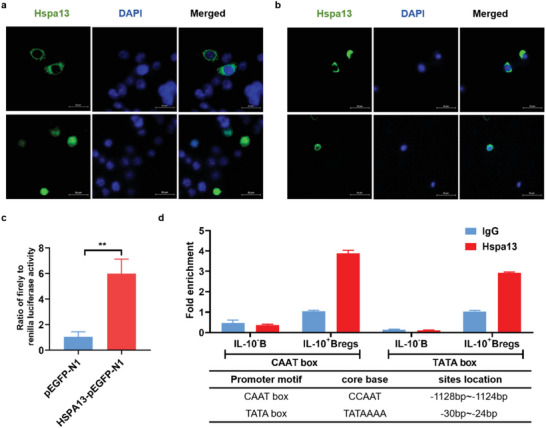
Hspa13 could bind to the IL‐10 promoter to activate IL‐10 transcription. a) Hspa13 was overexpressed in 293T cells via Hspa13‐pEGFP‐N1 vector transfection. The sub‐cellular localization of Hspa13 was observed in both the cellular nucleus and cytoplasm by confocal microscopy. b) B cells were stimulated for the induction of IL‐10 producing B cells. Hspa13 sub‐cellular localization in B cells via immunofluorescence staining was observed in both the cellular nucleus and cytoplasm by confocal microscopy. c) In the Hspa13‐pEGFP‐N1 and the IL‐10 promoter luciferase reporter plasmid transfected CHO cells, a significant induction of luciferase activity was observed (*n* = 3, ^**^ *p *< 0.01). d) The DNA sequences were pulled down by Hspa13 antibody from IL‐10^+^ Bregs and IL‐10^−^ B cells via CHIP experiments. The pulled‐down DNA sequences showed much higher fold enrichment for PCR amplification of the TATA box and CAAT box in the IL‐10 promoter sequence than in the control. Statistical analyses were performed by two‐tailed *t*‐tests between two groups, unless otherwise stated.

To investigate the potential interaction between Hspa13 and IL‐10, we first analyzed the subcellular localization of Hspa13. Full‐length cDNA of the mouse Hspa13 gene was cloned, and the recombinant vector Hspa13‐pEGFP‐N1 was constructed. The subcellular localization of Hspa13 was observed in the Hspa13‐pEGFP‐N1 transfected 293T cells using confocal microscopy. Interestingly, Hspa13 expression was observed in both the nucleus and the cytoplasm (**Figure** **3**a). After induction of IL‐10 producing B cells in vitro, Hspa13 sub‐cellular localization in B cells was assessed by immunofluorescence staining. Similarly, the results indicated that Hspa13 localized to the cytoplasm and nucleus of B cells (Figure 3b). Thus, we wondered whether Hspa13 may transfer into the nucleus to regulate IL‐10 transcriptional expression. We employed a luciferase reporter plasmid containing the IL‐10 promoter sequence (−1538/+64). A significant induction of luciferase activity was observed in Hspa13‐pEGFP‐N1 and IL‐10 promoter luciferase reporter plasmid transfected cells compared to that in the control cells (Figure 3c). Thus, Hspa13 functions as a potential transcription factor to improve the transcription of IL‐10. While analyzing the potential binding motif for HSPA13, the classical heat shock response elements (HSEs) including TATA box (−30 to −24 bp) and CAAT box (−1128 to −1124 bp) were found in the IL‐10 promoter. To further verify the binding of Hspa13 to the IL‐10 promoter, we obtained IL‐10^+^ Bregs and IL‐10^−^ B cells using the Bregs isolation kit and used specific primers for the IL‐10 promoter covering the TATA box and CAAT box separately to amplify the DNA sequences pulled down by the Hspa13 antibody. The pulled‐down DNA sequences showed a much higher fold enrichment for PCR amplification of the TATA box and CAAT box in the IL‐10 promoter sequence than in the control IgG (Figure 3d). Thus, the anti‐Hspa13 antibody pulled down a greater amount of the IL‐10 promoter sequence than the control IgG, implying that Hspa13 might directly bind to the IL‐10 promoter sequence via TATA box and CAAT box (Figure 3d).

### 2.4 Hspa13 Positive Cells are Largely Enriched in MZ B Cells to Regulate IL‐10 Production

**Figure 4:**
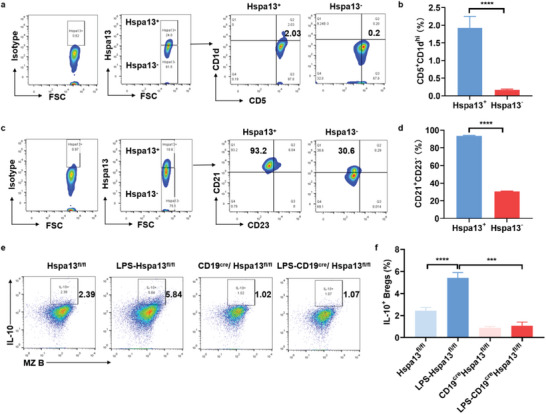
Hspa13 positive cells are largely enriched in the MZ B cells to regulate IL‐10 production. B cells were stimulated for the induction of IL‐10‐producing B cells in vitro. Hspa13 expression in B cells was analyzed using FACS via immunofluorescence staining. a) The flow plots, and b) percentages of CD5^+^CD1d^hi^ B cells were lowly enriched in Hspa13 positive cells by FACS analysis. c) The flow plots, and d) percentages of CD21^+^CD23^−^ B cells were largely enriched in Hspa13 positive cells by FACS analysis. e) MZ B cells were purified from spleens using the MZ B cells isolation kit. MZ B cells were treated using LPS and cell activation cocktail for the induction of IL‐10‐producing B cells. Genetic Hspa13 deficiency in the MZ B cells isolated from CD19^cre^Hspa13^fl/fl^ impaired the induction of IL‐10^+^ B cells proportion by FACS. f) The statistical percentage of IL‐10^+^ B cells in stimulated MZ B cells from CD19^cre^Hspa13^fl/fl^ mice decreased compared to that from Hspa13^fl/fl^ mice (*n* = 4, ^***^ *p *< 0.001, ^****^ *p *< 0.0001). Statistical analyses were performed by two‐tailed *t*‐tests between two groups, while two‐way ANOVA analysis were performed between multiple groups.

Previous studies have reported the identified Breg phenotypes, such as MZ B cells, T2‐MZP B cells, and CD5^+^CD1d^hi^ B cells. Acting as an important regulator of Bregs, it is wondering whether Hspa13 is selectively highly expressed in MZ, T2‐MZP B cells, or CD5^+^CD1d^hi^ B cells. With the induction of IL‐10^+^ Bregs in vitro, Hspa13 expression in B cells was analyzed by FACS. Hspa13 positive cells and negative cells were first gated on, and followed by phenotype analysis. Notably, Hspa13 positive cells were mostly enriched in CD21^+^CD23^−^ B cells (93.8%) compared to CD21^+^CD23^+^ (5.3%) and CD5^+^CD1d^hi^ B cells (1.9%) (**Figure** **4**a–d; Figure S1e, Supporting Information). Thus, Hspa13 is selectively and highly expressed in MZ B cells. Then, the purified MZ B cells from spleens using the MZ B cells isolation kit were treated with LPS and cell activation cocktail for the induction of IL‐10 producing B cells. The results showed a much lower proportion of IL‐10^+^ B cells in the MZ B cells isolated from CD19^cre^Hspa13^fl/fl^ mice than in cells isolated from Hspa13^fl/fl^ mice (Figure 4e,f). Taken together, these findings suggest that Hspa13 is largely enriched in the MZ B cells and regulates IL‐10 production in the MZ B cells.

### 2.5 Deficiency of Hspa13 in B Cells Impaired Their Function in Supporting Regulatory T Cells Differentiation

**Figure 5:**
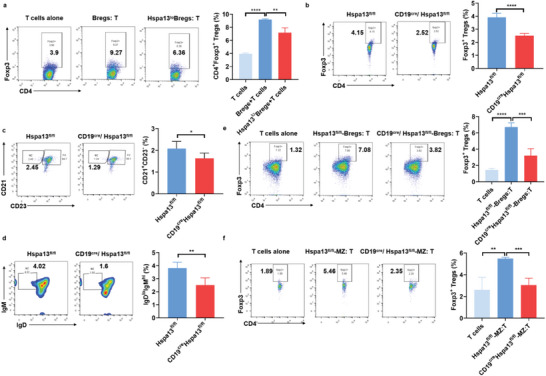
Deficiency of Hspa13 in B cells impaired their function in supporting regulatory T cells differentiation. a) B cells were transfected with Hspa13 shRNA retroviruses to knock down Hspa13 expression. Then, B cells were stimulated to induce the production of IL‐10^+^ Bregs, and co‐cultured with isolated CD4^+^ T cells in vitro. Co‐culture of CD4^+^ T cells with Hspa13 shRNA transfected B cells resulted in a decreased proportion of CD4^+^Foxp3^+^ Tregs compared to the control (*n* = 3, ^**^ *p *< 0.01, ^****^ *p *< 0.0001). b) The proportion of spleen CD4^+^Foxp3^+^ Tregs significantly reduced in the CD19^cre^/Hspa13^fl/fl^ mice compared to that in the Hspa13^fl/fl^ mice via FACS analysis (*n* = 6, ^****^ *p *< 0.0001). The percentages of c) CD21^+^CD23^−^, or d) IgD^lo^IgM^hi^ MZ B cells in the spleens of CD19^cre^/Hspa13^fl/fl^ mice decreased compared to the Hspa13^fl/fl^ mice by FACS analysis (*n* = 4, ^*^ *p *< 0.05, ^**^ *p *< 0.01). e) B cells were stimulated for the induction of IL‐10^+^ Bregs, and co‐cultured with isolated CD4^+^ T cells in vitro. Co‐culture of CD4^+^ T cells with stimulated B cells isolated from CD19^cre^/Hspa13^fl/fl^ mice resulted in a dramatic decrease of CD4^+^Foxp3^+^ Tregs compared to that from Hspa13^fl/fl^ mice (*n* = 4, ^***^ *p *< 0.001, ^****^ *p *< 0.0001). f) MZ B cells were isolated and stimulated for the induction of IL‐10^+^ Bregs, and co‐cultured with isolated CD4^+^ T cells in vitro. Co‐culture of CD4^+^ T cells with stimulated MZ B cells purified from CD19^cre^/Hspa13^fl/fl^ mice led to a significant reduction of CD4^+^Foxp3^+^ Tregs than that from the Hspa13^fl/fl^ mice (*n* = 4, ^*^ *p *< 0.05, ^***^ *p *< 0.001). Statistical analyses were performed by two‐tailed *t*‐tests between two groups, while two‐way ANOVA analysis were performed between multiple groups.

Regulatory T cells (Tregs) play a critical role in maintaining immune homeostasis. Bregs could exert their regulatory function by promoting the differentiation of resting CD4^+^ T cells into Tregs.^[^ ^29^, ^30^ ^]^ To evaluate the effect of Hspa13 deficiency on Breg function, B cells transfected with Hspa13 shRNA retroviruses were treated to induce the production of IL‐10^+^ Bregs, and then co‐cultured with isolated CD4^+^ T cells in vitro. Compared to CD4^+^ T cells cultured alone, co‐culture of CD4^+^ T cells with Bregs significantly increased the percentage of CD4^+^Foxp3^+^ Tregs. However, co‐culture of CD4^+^ T cells with Hspa13 shRNA‐transfected Bregs resulted in a decreased proportion of CD4^+^Foxp3^+^ Tregs compared to co‐culture with control Bregs (**Figure** **5**a). In the CD19^cre^/Hspa13^fl/fl^ mice, the proportion of splenic CD4^+^Foxp3^+^ Tregs was significantly reduced than that in the Hspa13^fl/fl^ mice (Figure 5b). And, the IL‐10 supplementary could partially restore the CD4^+^Foxp3^+^ Tregs proportion in spleens of CD19^cre^/Hspa13^fl/fl^ mice (Figure S2, Supporting Information). The results further verified that reduced Tregs proportion was affected by the impaired Bregs function caused by Hspa13 deficiency. Meanwhile, the percentages of CD21^+^CD23^−^ or IgD^lo^IgM^hi^ MZ B cells in the spleens of CD19^cre^/Hspa13^fl/fl^ mice were decreased compared to that in Hspa13^fl/fl^ mice (Figure 5c,d). The results remind that Hspa13 deficiency in B cells could affect MZ B cells and Tregs differentiation. Additionally, co‐culture of CD4^+^ T cells with stimulated B cells isolated from CD19^cre^/Hspa13^fl/fl^ mice resulted in a dramatic decrease of CD4^+^Foxp3^+^ Tregs compared to the Hspa13^fl/fl^ mice (Figure 5e). Similarly, co‐culture of CD4^+^ T cells with stimulated MZ B cells purified from CD19^cre^/Hspa13^fl/fl^ mice led to a significant reduction of CD4^+^Foxp3^+^ Tregs compared to the Hspa13^fl/fl^ mice (Figure 5f). Taken together, these results indicate that Hspa13 deficiency impairs the regulatory function of Bregs.

### 2.6 Functional Decline of Bregs was Accompanied with Decreased Hspa13 Expression in Bregs as Well as MZ B Cells

**Figure 6:**
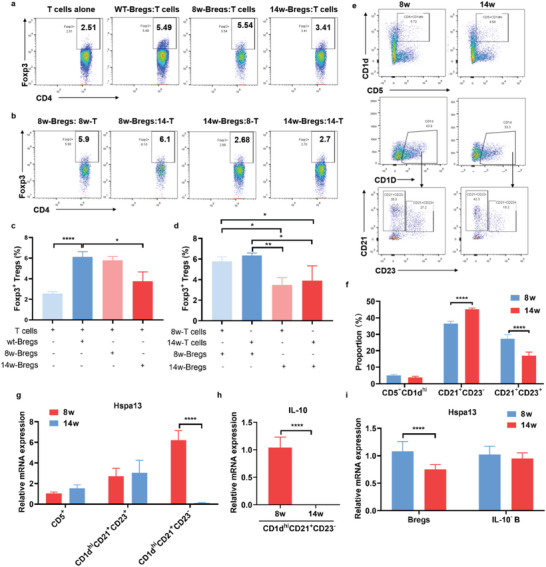
The functional decline of Bregs was accompanied with decreased Hspa13 expression in Bregs as well as MZ B cells. B cells isolated from MRL/lpr mice aged at 8 and 14 weeks were stimulated to induce the production of IL‐10^+^ Bregs, and then co‐cultured with CD4^+^ T cells obtained from C57BL/6J mice. Bregs function on supporting Tregs differentiation was impaired in MRL/lpr mice aged at 14 weeks but not in mice at 8 weeks. The a) flow plots, and c) statistical percentages of Tregs were reduced using FACS analysis (*n* = 4, ^*^ *p *< 0.05, ^****^ *p *< 0.0001). B cells isolated from 8w‐MRL/lpr mice and 14w‐MRL/lpr mice were stimulated to induce the production of IL‐10^+^ Bregs, and then co‐cultured with CD4^+^ T cells obtained from 8w‐MRL/lpr mice or 14w‐MRL/lpr mice separately. Tregs differentiation ability is not affected in 14w‐MRL/lpr mice, but stimulated B cells function on supporting Tregs differentiation is partially weakened in 14w‐MRL/lpr mice. The b) flow plots, and d) statistical percentages of Tregs were decreased using FACS analysis (*n* = 4, ^*^ *p *< 0.05, ^**^ *p *< 0.01). e) T2‐MZP, MZ B cells, and CD5^+^CD1d^hi^ B cells were sorted from 8w‐MRL/lpr mice and 14w‐MRL/lpr mice by FACS. f) The percentages of MZ B cells, T2‐MZP cells, and CD5^+^CD1d^hi^ B cells were statistically calculated in MRL/lpr mice (*n* = 4, ^****^ *p *< 0.0001). g) Relative mRNA expression of Hspa13 was detected in T2‐MZP, MZ B cells, and CD5^+^CD1d^hi^ B cells by real‐time PCR analysis (*n* = 6, ^****^ *p *< 0.0001). h) IL‐10 mRNA expression was reduced in MZ B cells of 14w‐MRL/lpr mice than that in 8w‐MRL/lpr mice via real‐time PCR analysis (*n* = 6, ^****^ *p *< 0.0001). i) Relative mRNA expression of Hspa13 decreased in IL‐10^+^ Bregs from 14w‐MRL/lpr mice than that from 8w‐MRL/lpr mice (*n* = 6, ^****^ *p *< 0.0001). Statistical analyses were performed by two‐tailed *t*‐tests between two groups, while two‐way ANOVA analysis were performed between multiple groups.

Previous studies reported that SLE patients showed a functional decline of the CD24^high^CD38^high^ Bregs in inhibiting the production of IFN‐γ and TNF‐α by CD4^+^ T cells after CD40 stimulation.^[^ ^13^, ^31^ ^]^ Due to the critical role of Tregs in maintaining self‐tolerance and preventing excessive immune responses, a deficiency in the quantity or function of Tregs could lead to immune tolerance loss against self‐antigens in autoimmune diseases.^[^ ^32^ ^]^ To further explore Hspa13 function in SLE, we utilized the autoimmune lupus‐prone MRL/Mpr‐Fas^lpr^ (MRL/lpr) mouse strain that spontaneously develops lupus‐like disease. A decrease in the frequency of CD4^+^Foxp3^+^ Tregs has been reported in diseased MRL/lpr mice.^[^ ^33^ ^]^ We also verified that the frequency of splenic CD4^+^Foxp3^+^ Tregs was reduced in MRL/lpr mice at the active‐disease stage (aged at 12–14 weeks) compared with MRL/lpr mice at the pre‐disease stage (aged at 6–8 weeks) (Figure S3a, Supporting Information). The loss of Tregs in the lupus mice might ascribe to the capacity impairment of the immune components like Bregs in supporting Tregs differentiation or the disability of Tregs differentiation. To explore whether decreased Treg frequency directly correlates with dysregulated Bregs, B cells isolated from MRL/lpr mice in the pre‐disease and active‐disease stages were stimulated to induce the production of IL‐10^+^ Bregs and then co‐cultured with CD4^+^ T cells obtained from C57BL/6J mice. We found that the co‐culture of CD4^+^ T cells with Bregs from 8w‐MRL/lpr mice increased the CD4^+^Foxp3^+^ Tregs proportion as Bregs obtained from C57BL/6J mice compared to the T cells cultured alone (**Figure** **6**a,c). However, the co‐culture of CD4^+^ T cells with Bregs from 14w‐MRL/lpr mice failed to increase the CD4^+^Foxp3^+^ Tregs proportion (Figure 6a,c). The results suggest that Bregs function on supporting Treg differentiation was impaired in active lupus‐prone MRL/lpr mice. Furthermore, Bregs obtained from 8w‐MRL/lpr mice could support CD4^+^ T cells enriched from both 14w‐MRL/lpr mice and 8w‐MRL/lpr mice differentiating into CD4^+^Foxp3^+^ Tregs (Figure 6b,d). Conversely, Bregs from 14w‐MRL/lpr mice displayed a weakened ability to support the differentiation of CD4^+^ T cells from both 8w‐MRL/lpr mice and 14w‐MRL/lpr mice into Tregs (Figure 6b,d). Thus, Tregs differentiation ability was not affected, but Bregs function on supporting Tregs differentiation was partially weakened in lupus MRL/lpr mice. Next, we analyzed and sorted Breg subsets, including T2‐MZP, MZ B cells, and CD5^+^CD1d^hi^ B cells from 8w‐MRL/lpr mice and 14w‐MRL/lpr mice by FACS (Figure 6e,f). Interestingly, relative Hspa13 mRNA expression was much higher in the MZ B cells than in the T2‐MZP B cells and CD5^+^CD1d^hi^ B cells isolated from 8w‐MRL/lpr mice at the pre‐disease stage. Moreover, relative Hspa13 mRNA selectively decreased in MZ B cells rather than in T2‐MZP B cells or CD5^+^CD1d^hi^ B cells obtained from 14w‐MRL/lpr mice than those from 8w‐MRL/lpr mice (Figure 6g). Moreover, IL‐10 mRNA expression decreased in MZ B cells enriched from 14w‐MRL/lpr mice compared with those from 8w‐MRL/lpr mice (Figure 6h). Additionally, we purified IL‐10^+^ Bregs and IL‐10^−^ B cells from the spleens of 8w‐MRL/lpr mice and 14w‐MRL/lpr mice separately. The relative Hspa13 mRNA expression was obviously decreased in IL‐10^+^ Bregs from 14w‐MRL/lpr mice than that in the 8w‐MRL/lpr mice, but not in the IL‐10^−^ B cells (Figure 6i). Accordingly, the above findings verified that the decline in Breg function supporting Tregs differentiation was accompanied by decreased Hspa13 expression.

### 2.7 Hspa13 Deficiency in B Cells Impaired Their Immune Regulatory Function in Attenuating Renal Pathology of MRL/lpr Mice

**Figure 7:**
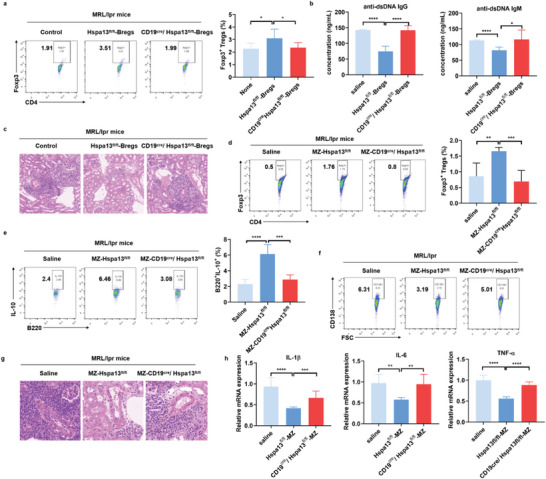
Hspa13 deficiency in B cells impaired their immune regulatory function in attenuating renal pathology of MRL/lpr mice. The enriched Bregs obtained from Hspa13^fl/fl^ mice or CD19^cre^/Hspa13^fl/fl^ mice were adoptively transferred into 12w‐MRL/lpr mice in the active‐disease stage. Transfer of enriched Breg cells from CD19^cre^/Hspa13^fl/fl^ mice displayed impaired capacity in a) increasing the CD4^+^Foxp3^+^ Tregs frequency, b) reducing serum anti‐dsDNA autoantibodies levels, and c) ameliorating lupus nephritis especially with less severe glomerular pathology and inflammatory cell infiltration in renal arterioles and perivascular interstitium compared to the Hspa13^fl/fl^ mice (*n* = 6, ^*^ *p *< 0.05, ^****^ *p *< 0.0001). Transfer of stimulated MZ B cells from CD19^cre^/Hspa13^fl/fl^ mice failed to d) increase the CD4^+^Foxp3^+^ Tregs, e) IL‐10^+^ Bregs frequency, f) decrease CD138^+^ plasma cells proportion, g) alleviate renal inflammatory cell infiltration, and h) reduce renal inflammatory factors compared to the Hspa13^fl/fl^ mice (*n* = 6, ^**^ *p *< 0.01, ^***^ *p *< 0.001, and ^****^ *p *< 0.0001). Statistical analyses were performed by two‐tailed t‐tests between two groups, while two‐way ANOVA analysis were performed between multiple groups.

The reduced frequency of splenic CD4^+^Foxp3^+^ Tregs, increased serum anti‐dsDNA autoantibody levels, and aggravated renal pathological changes were observed in 14w‐MRL/lpr mice at the early disease stage compared with 8w‐MRL/lpr mice at the pre‐disease stage (Figure S3a–d, Supporting Information). To directly address the effects of Hspa13 deficiency on Bregs function in vivo, we adoptively transferred enriched Bregs obtained from Hspa13^fl/fl^ or CD19^cre^/Hspa13^fl/fl^ mice into MRL/lpr mice in active‐disease stage. Treg frequency, autoantibodies, and renal pathological changes were detected to assess Hspa13 function in determining Breg function and improving lupus pathology. Compared to saline‐infused MRL/lpr mice, transfer of stimulated B cells for Breg induction from Hspa13^fl/fl^ mice resulted in significant beneficial effects by increasing the CD4^+^Foxp3^+^ Tregs proportion in spleen and relieving renal pathological abnormalities in glomerulus, tubule‐interstitium, and vessels. While, transfer of enriched Breg cells from CD19^cre^/Hspa13^fl/fl^ mice displayed the impaired capacity in increasing the CD4^+^Foxp3^+^ Tregs frequency, reducing serum anti‐dsDNA autoantibody levels, and ameliorating lupus nephritis especially with less severe glomerular pathology and inflammatory cell infiltration in renal arterioles and perivascular interstitium when compared with Breg cells from Hspa13^fl/fl^ mice (**Figure** **7**a–c). Similarly, transfer of stimulated MZ B cells for Breg induction from CD19^cre^/Hspa13^fl/fl^ mice also failed to increase the CD4^+^Foxp3^+^ Tregs and IL‐10^+^ Bregs frequency, decrease the CD138^+^ plasma cell proportion, and alleviate inflammatory factors levels and renal pathological changes compared with those from Hspa13^fl/fl^ mice (Figure 7d–h). Therefore, our findings support the idea that Hspa13 deficiency in MZ B cells impaired their immune regulatory function in attenuating lupus pathology of MRL/lpr mice.

## 3 Discussion

Studies have reported that the number and function of IL‐10 producing Bregs are impaired in murine lupus and human SLE. However, the mechanisms by which Bregs are functionally altered and affected in lupus pathology remains unclear. In this study, we identified Hspa13 as a novel positive regulator of IL‐10 producing Bregs and demonstrated that Hspa13 deficiency in MZ B cells contributed to lupus disease pathology by reducing Tregs. Thus, our results illustrate a vital role of Hspa13 in promoting IL‐10 producing Bregs production and regulatory function, and partially elucidates the reason for the impaired regulatory function of the MZ B cells in lupus‐prone mice.

Several described different phenotypes of IL‐10‐producing Bregs, including T2‐MZP B cells, CD5^+^CD1d^hi^ B cells, MZ B cells, Tim‐1^+^ B cells, CD138^+^ plasma cells, and plasmablasts, suggest that multiple subsets of B cells at different developmental stages have the potential to differentiate into IL‐10‐producing Breg cells.^[^ ^4^, ^29^, ^34^ ^]^ Together with the findings of B cells acquiring a regulatory phenotype in response to certain stimuli, it supports the idea that the induction or differentiation of Bregs should correlate with some reactive or inducible factors evoked by the right environmental stimuli. HSPs are highly conserved proteins, most of which are stress‐inducible to play the cytoprotective roles while exposed to various stressful conditions.^[^ ^35^ ^]^ Our results proved that Hspa13 was quickly induced while inducing Bregs production, suggesting that Hspa13 is an inducible factor in response to stimuli. Moreover, our findings further verified the positive regulation of Hspa13 on IL‐10 production and the immune regulatory function of B cells. Thus, Hspa13 could function as a reactive and functional factor in IL‐10‐producing Bregs.

Studies reported that HSPs could act as immunogenic proteins and involve in the pathogenesis of inflammatory and autoimmune diseases.^[^ ^17^, ^36^, ^37^ ^]^ Moreover, the anti‐inflammatory effects and immunosuppressive function of Hsp70 have been proposed in both innate and adaptive immunity.^[^ ^21^ ^]^ For example, extracellular Hsp70 can modulate cytokine production and innate cell function by interacting with receptors on cells such as monocytes, dendritic cells (DCs) and myeloid‐derived suppressor cells (MDSCs).^[^ ^38^, ^39^ ^]^ Besides, the presentation of Hsp70 peptides in MHC molecules also leads to the induction of Hsp‐specific Tregs.^[^ ^40^ ^]^ Currently, as a member of the HSP70 family, the biological functions of Hspa13 in the regulation of immune responses are limited. It has been reported that Hspa13 restricts viral infection by modulating the type I interferon antiviral pathway and the NLRP3 inflammasome, revealing the antiviral effects of Hspa13.^[^ ^25^ ^]^ In one of our previous studies, Hspa13 was proved to be critical for plasma cell development.^[^ ^26^ ^]^ In this study, the findings reported the novel functions of Hspa13 in regulating IL‐10 production and the immune regulatory function of B cells by supporting Tregs differentiation. Therefore, our results provide new evidence for the function of Hspa13 in regulating adaptive immune responses by enhancing the production of the anti‐inflammatory cytokine IL‐10 in B cells and improving the regulatory function of Bregs.

The classical roles of HSPs function as intracellular chaperones in maintaining proteostasis involved in protein folding, protein trafficking, and protein complex assembly/disassembly.^[^ ^17^ ^]^ Increasing number of studies have reported that some HSPs can be secreted to the cell exterior in response to stress and are generally regarded as danger signaling biomarkers.^[^ ^41^ ^]^ Studies have suggested that Hspa13 acts as a scaffolding factor to regulate endoplasmic reticulum and cytosolic proteostasis by modulating protein translocation and stability.^[^ ^22^, ^23^, ^24^ ^]^ In this study, we found that Hspa13 can directly bind to the IL‐10 promoter sequence and act as a potential transcription factor in the nucleus. Thus, the findings newly reveal the subcellular localization of Hspa13 in the nucleus and identify Hspa13 as a novel IL‐10 regulator.

Innate‐like MZ B cells, located in the microanatomical area of the splenic marginal zone, participate in a quick response to blood‐borne pathogens and the production of natural antibodies. Other important features of the MZ B cells include continuous antigen transport between the marginal zone and follicle and presentation of antigens to CD4^+^ T cells.^[^ ^42^ ^]^ The expanded MZ B cell compartment, which has the ability for self‐antigen presentation and autoantibody secretion, has been demonstrated in several autoimmune conditions.^[^ ^42^ ^]^ Notably, MZ B cells can also differentiate into IL‐10 producing B cells and regulate immune responses, which may contribute to the regulation of systemic autoimmunity in mice.^[^ ^5^, ^6^, ^43^ ^]^ Thus, MZ B cells can use both their canonical B cell features as well as their more innate‐like qualities, such as being hyper‐responsive to TLR‐stimulation in their quick response to antigenic stimulation. Dysregulated MZ B cells contribute to disrupting tolerance and play pathogenic roles in autoimmune diseases. However, the potential immunoregulatory function of MZ B cells is neglected in lupus. Recently, a phenotypic analysis of IL‐10 producing regulatory B cells by single‐cell RNA sequencing was performed in lupus‐prone mice, revealing a loss of marginal zone lineage Bregs in active‐disease lupus‐prone mice.^[^ ^44^ ^]^ In the present study, we proved the loss of IL‐10 producing MZ B cells as well as the immune regulatory function in regulating Treg induction in the active‐disease stage of the lupus mice. Importantly, we proved that the deficiency of Hspa13 contributed to impaired IL‐10 production and immune regulatory function of MZ B cells, both in vitro and in lupus mice. Therefore, these results explain the unique protective role of Hspa13 in determining MZ regulatory function and affecting the pathogenesis of lupus.

In conclusion, we successfully identified Hspa13 as a novel transcriptional regulator of IL‐10 in B cells, exerting the regulatory effect by supporting Treg differentiation. Importantly, the decline of Bregs function supporting Treg differentiation was accompanied by decreased Hspa13 expression in lupus MRL/lpr mice. Hspa13 deficiency in B cells, especially MZ B cells, impaired their immune regulatory function in attenuating renal pathology of MRL/lpr mice. Thus, our results reveal a novel role of Hspa13 in regulating MZ B cell function and affecting lupus pathogenesis.

## 4 Experimental Section

### Animals

The IL‐10‐EGFP reporter tiger mice were kindly provided by Professor Yinxiang Wei. Tiger mice were generated by inserting internal ribosome entry site (IRES) green fluorescent protein (GFP) immediately before the polyadenylation site of the IL‐10 gene. Thus, designated tiger (interleukin‐ten ires gfp‐enhanced reporter) mice provide a valuable tool for in vivo IL‐10 analysis and detection.^[^ ^27^, ^45^ ^]^ The floxed Hspa13 (Hspa13^fl/fl^) mice and CD19^cre^ mice in a B6 background were generated by Shanghai Biomodel Organism Science & Technology Development Co., Ltd. (Shanghai, China). To achieve specific Hspa13 deletion in B cells, Hspa13^fl/fl^ mice were crossed with CD19^cre^ mice to generate CD19^cre^Hspa13^fl/fl^ (Hspa13 cKO) mice. C57BL/6 and MRL/lpr mice (MRL/MpJ‐Faslpr) were purchased from SPF Biotechnology Co., Ltd (Beijing, China). Female MRL/lpr mice suffer earlier and more severe disease syndromes than male MRL/lpr mice, similar to human patients with lupus. In the present study, female MRL/lpr mice aged at 6–8 weeks (the pre‐disease stage) and those aged at 12–14 weeks (the active disease stage) were used to represent the different disease stages. Mice are randomly assigned to the control and experimental group based on their body weights. All mice used in each group (*n* = 6/group) were bred in animal facilities under specific pathogen‐free conditions with a 12:12 h light‐dark cycle. The care, use, and treatment of mice in this study were in strict agreement with the international guidelines for the care and use of laboratory animals. This study was approved by the Animal Ethics Committee of Beijing Institute of Basic Medical Sciences (IACUC‐DWZX‐2021‐692).

### Cell Sorting

IL‐10‐positive or IL‐10‐negative B220^+^ cells isolated from IL‐10‐EGFP reporter tiger mice were acquired using the FACSAria system (BD Biosciences, San Jose, USA), depending on GFP expression in gated live B220 positive lymphocyte cells. B220^+^ B cells, MZ B cells, and CD4^+^T cells were isolated by magnetic‐bead purification according to the manufacturer's instructions using mouse B220^+^ microbeads (130‐049‐501, Miltenyi Biotec, Bergisch Gladbach, Germany), marginal zone and follicular B cell isolation kit (130‐100‐366, Miltenyi Biotec, Bergisch Gladbach, Germany), and CD4 microbeads (130‐117‐043, Miltenyi Biotec, Bergisch Gladbach, Germany), separately.

### IL‐10‐Producing Bregs Induction, Purification, and Adoptive Transfer

Enriched B220^+^ B cells or MZ B cells were planted in complete RPIM 1640 medium (R8758, Sigma–Aldrich, San Louis, USA) with 10% fetal bovine serum (FBS) (A5256701, Gibco, New York, USA) and maintained in standard cell culture environment (95% humidity, 5% CO2 at 37 °C). For IL‐10‐producing Bregs induction, the cultured B220^+^ B cells or MZ B cells were stimulated for 72 h with 10 µg mL^−1^ LPS (*Escherichia coli* 055:B5, L6529, Sigma–Aldrich, San Louis, USA), followed by stimulation with cell activation cocktail (423301, Biolegend, San Diego, USA) during the last 5 h. For separation of IL‐10‐producing Bregs, regulatory B cell isolation kit (130‐095‐873, Miltenyi Biotec, Bergisch Gladbach, Germany) was used. Briefly, stimulated B220^+^ B cells or MZ B cells were labeled and incubated with regulatory B cell catch reagent for 45 min. After removing the supernatant, the cells were resuspended and labeled with a regulatory B cell detection antibody. Anti‐PE microbeads were mixed into the cell suspension before magnetic separation on the LS columns to enrich IL‐10 secreting B cells through positive selection. For adoptive transfer, each lupus‐prone mouse was injected with isolated IL‐10‐producing Breg cells (one million) through the tail vein. Two injections were administered at age of 12 and 13 weeks for MRL/lpr mice (active disease stage). The treatment efficiency of IL‐10‐producing Bregs was analyzed 2 weeks after adoptive transfer.

### Cell Co‐Culture

For the analysis of CD4^+^ T cell proliferation and Treg differentiation, isolated CD4^+^T cells were cultured alone or co‐cultured (1:1) with purified LPS‐induced Bregs from isolated splenic B220^+^ B cells, MZ B cells of Hspa13^fl/fl^ mice, or CD19^cre^Hspa13^fl/fl^ mice for 72 h. The following CD4^+^Foxp3^+^ Tregs frequency were detected by flow cytometric analysis.

### Overexpression of Hspa13

Hspa13‐pEGFP‐N1 plasmid was generated in the laboratory. The mouse Hspa13 (GenBank Accession number NM_030201) sequence was derived via PCR‐based amplification from LPS stimulated B cells and subcloned into the pEGFP‐N1 vector. Over‐expression of Hspa13 in Chinese hamster ovary (CHO) cells (1101HAM‐PUMC000116, Cell Resource Center, Institute of Basic Medical Sciences, Beijing, China) was achieved via Hspa13‐pEGFP‐N1 plasmid transfection. Briefly, CHO cells (1 × 10^6^ cells mL^−1^) were cultured for 1 day in 6‐well plates in DMEM (11965092, Gibco, New York, USA) containing 10% FBS in the presence of 1 mL empty vector or Hspa13‐pEGFP‐N1 plasmid. The infected cells were washed and cultured for another 3 days in 6‐well plates in the presence of 10 µg mL^−1^ LPS. On day 3, cells were collected for further experiments.

### Knockdown of Hspa13

Isolated B cells from the spleen were cultured in 24‐well plates (1 ×10^6^ cells mL^−1^, total volume 2 mL) in RPMI 1640 medium (R8758, Sigma–Aldrich, San Louis, USA) containing 10% FBS, 2 mm glutamine, penicillin (100 IU mL^−1^), streptomycin (100 mg mL^−1^), and 50 mm 2‐ME in the presence of 100 µL control lentivirus or sh‐Hspa13 lentivirus (sc‐153884‐V, Santa Cruz Biotechnology, Santa Cruz, USA). The infected cells were stimulated with 10 ug mL^−1^ LPS for 72 h, with cell activation cocktail (423301, Biolegend, San Diego, USA) during the last 5 h of incubation.

### Flow Cytometric Analysis

All cell experiments were strictly prepared on ice, unless otherwise stated in other specific procedures. Splenocytes (1×10^6^ cells/sample) were washed with fluorescence‐activated cell sorting (FACS) staining buffer (phosphate‐buffered saline (PBS) (G4202, Servicebio, Wuhan, China), 2% fetal bovine serum (FBS) (C04001‐500, VivaCell BIOSCIENCES, Shanghai, China), 0.1% sodium azide). Briefly, single‐cell suspensions were blocked on ice with anti‐Fc receptor Ab (clone 2.4G2, 569324, BD Biosciences, San Jose, USA), stained for 30 min in the dark with fluorochrome‐conjugated antibodies, washed with FACS washing buffer, and finally analyzed using a BD FACS Aria II flow cytometer (BD Biosciences, San Jose, USA). For intracellular staining, cells were collected and fixed for 50 min with the fixation buffer (420801, Biolegend, San Diego, USA) and washed with intracellular staining permeabilization wash buffer (421002, Biolegend, San Diego, USA). After washing, the fixed cells were stained. The samples were filtered before analysis or cell sorting to remove any clumps. The following antibodies were used: FITC‐conjugated anti‐mouse CD1d (123507, BioLegend, San Diego, CA, USA), PE‐CD21 (123409, BioLegend), or APC/Cyanine7‐conjugated anti‐mouse CD23 (101629, BioLegend), PerCP‐conjugated anti‐mouse B220 (103233, BioLegend), PerCP‐conjugated anti‐mouse CD4 (100537, BioLegend), APC‐conjugated anti‐mouse B220 (103211, Biolegend), PE/Cyanine7‐conjugated anti‐mouse IL‐10 (505025, Biolegend), and PE‐conjugated anti‐mouse foxp3 (118904, BioLegend). Data collection and analyses were performed on a FACS Calibur flow cytometer using the CellQuest software.

### Quantitative PCR (qPCR) Analysis

Total RNA was extracted from cells lysed with TRIzol (T9424, Sigma–Aldrich, St. Louis, USA) according to the manufacturer's instructions. Reverse transcription reactions were carried out to obtain the cDNA products using the Thermo ScriptTM RT‐PCR system (M1631, Thermo Fisher Scientific, Waltham, USA). The qPCR was employed to quantify target gene expression in the cDNA samples. The amplification of target genes was performed using the standard protocol of a Bio‐Rad T100TM Thermal Cycler (1861096, Bio‐Rad, Hercules, CA, USA). Mouse gene expression was normalized to the levels of the β‐actin gene.

### Western Blot

The harvested fresh cell samples were lysed with ice‐cold lysis buffer supplemented with protease inhibitors (04693116001; Roche, Basel, Switzerland). Proteins in the cell lysate were transferred to a polyvinylidene fluoride membrane (PVDF) (ISEQ00010, Millipore, Billerica, USA) by 10% SDS‐PAGE, blocked with 5% skim milk, and incubated with primary antibody at 4 °C overnight. Primary antibodies against Hspa13 (12667‐2‐AP, Proteintech, Chicago, USA) and β‐actin (20536‐1‐AP, Proteintech, Chicago, USA) were used. The membrane was incubated with the corresponding horseradish peroxidase (HRP)‐conjugated secondary antibodies for 1 h at room temperature. After extensive wash for three times, the bands were detected using an ECL detection system (4600SF, Tanon Science & Technology Co., Ltd, Shanghai, China).

### Luciferase Reporter Assay

A luciferase reporter vector containing the IL‐10 promoter sequence was obtained from Addgene (pGL2B ‐1538/+64, #24942, Massachusetts, USA). CHO cells (1101HAM‐PUMC000116, Cell Resource Center, Institute of Basic Medical Sciences, Beijing, China) were co‐transfected with 100 ng luciferase reporter plasmid, 10 ng thymidine kinase promoter‐Renilla luciferase reporter plasmid, and Hspa13‐pEGFP‐N1 plasmid or a control vector. After 48 h, the luciferase activity was detected via the Dual‐Luciferase Reporter Assay System (E10910, Promega, Madison, USA) according to the manufacturer's instructions.

### Immunofluorescence Assay

For analyzing the subcellular location, Hspa13 was overexpressed in 293T cells or induced in isolated B cells. Over‐expression of Hspa13 in 293T cells (SCSP‐502, National Collection of Authenticated Cell Cultures, Shanghai, China) was carried out via Hspa13‐pEGFP‐N1 plasmid transfection. The inducible expression of Hspa13 in B cells was conducted via stimulation with LPS and cell activation cocktail, as described above. Briefly, the transfected 293T cells or stimulated B cells were fixed in 4% formaldehyde (G1101, Servicebio, Wuhan, China) and permeabilized with 0.3% Triton X‐100 (T8787, Sigma‐Aldrich, San Louis, USA). Cells were then blocked with 5% horse serum (C2510‐0500, VivaCell BIOSCIENCES, Shanghai, China) in PBS‐T, followed by overnight incubation with an anti‐Hspa13 antibody (1:100) (ab219172, Abcam, Cambridge, UK). Then, cells were washed and incubated with Alexa Fluor 488 conjugate (A11008, Invitrogen, Carlsbad, USA). Finally, the cells were counterstained with DAPI (C0060; Solarbio, Beijing, China) at room temperature. Stained cells were visualized using a confocal microscope (IX73; Olympus, Tokyo, Japan).

### Chromatin Immunoprecipitation (ChIP) Assay

ChIP assay was performed using an Enzymatic Chromatin IP Kit (9002, Cell Signal Technology, Boston, USA) according to the manufacturer's protocol. ChIP primers were designed to specifically amplify the regions covering the putative Hspa13 responsive elements within the mouse IL‐10 promoter region. Sequences of the primers used in ChIP assays were as follows: IL‐10: forward: 5′‐attgttcagtcagtgaatgtacag‐3′, reverse: 5′‐actacacaggtcggaatttatgta‐3′.

### Determination of Cytokine and Antibody Levels by Enzyme Linked Immunosorbent Assay (ELISA)

The concentration of IL‐10 in the cell culture supernatants or serum was measured using ELISA kits (SEA056Mu; CLOUND‐CLONE CORP, Wuhan, China). The concentrations of serum anti‐dsDNA IgM (SU‐BN21736, Chengzhi Kewei Biotechnology Co., Ltd., Beijing, China) and anti‐dsDNA IgG antibodies (SU‐BN21735, Chengzhi Kewei Biotechnology Co., Ltd.) were detected using ELISA kits. The assay procedures were performed according to the protocol provided by the manufacturer.

### Renal Hematoxylin eosin (HE) Staining

Kidney tissues were fixed with 10% neutral‐buffered formalin (HT501640, Sigma–Aldrich, St. Louis, USA) and embedded in paraffin. Paraffin sections were baked, dewaxed, hydrated, and stained with hematoxylin and eosin. After washing, the sections were dehydrated in a gradient of ethanol solution, made transparent in dimethylbenzene, mounted, and observed under a microscope (CX41; Olympus, Japan).

### Statistical Analysis

Results are presented as mean ± standard deviation. Differences between the experimental and control groups were evaluated by Student's *t*‐test or two‐way ANOVA. All statistical analyses were performed using the Prism v8.0 software (GraphPad, La Jolla, CA, USA) and the results were considered statistically significant at ^*^ *p* < .05, ^**^ *p* < .01, ^***^ *p* < .001, and ^****^ *p* < .001.

## Conflict of Interest

The authors declare no conflict of interest.

## Author Contributions

C.X. and R.W. conceptualized and coordinated the project. H.C. contributed to animal breeding. G. L., X. L., and X. H. performed most of the experiments and data analysis. R. W. and L. S. provided suggestions and guidance of this study. C.X. and R.W. wrote and revised the manuscript. All authors discussed the results and commented on the manuscript.

## Supporting information



Supporting Information

## Data Availability

The data that support the findings of this study are available from the corresponding author upon reasonable request.
