# Supplementary material for: Hspa13 Deficiency Impaired Marginal Zone B Cells Regulatory Function and Contributed to Lupus Pathogenesis
Source: Adv Sci (Weinh). 2024 Dec 31;12(8):2413144. doi: 10.1002/advs.202413144 (PMC11848637; doi:10.1002/advs.202413144)
Supplement: Supplementary file 1 — Supporting Information [file ADVS-12-2413144-s001.docx]

Supporting Information

Hspa13 Deficiency Impaired Marginal Zone B cells Regulatory Function and Contributed to Lupus Pathogenesis

*Chen Xing*, Haoran Cui, Ge Li, Xiaoling Liu, Kun Liu, Qing Wen, Xin Huang*, Renxi Wang*, Lun Song**

Figures S1-3

Supporting Information

Figures S1


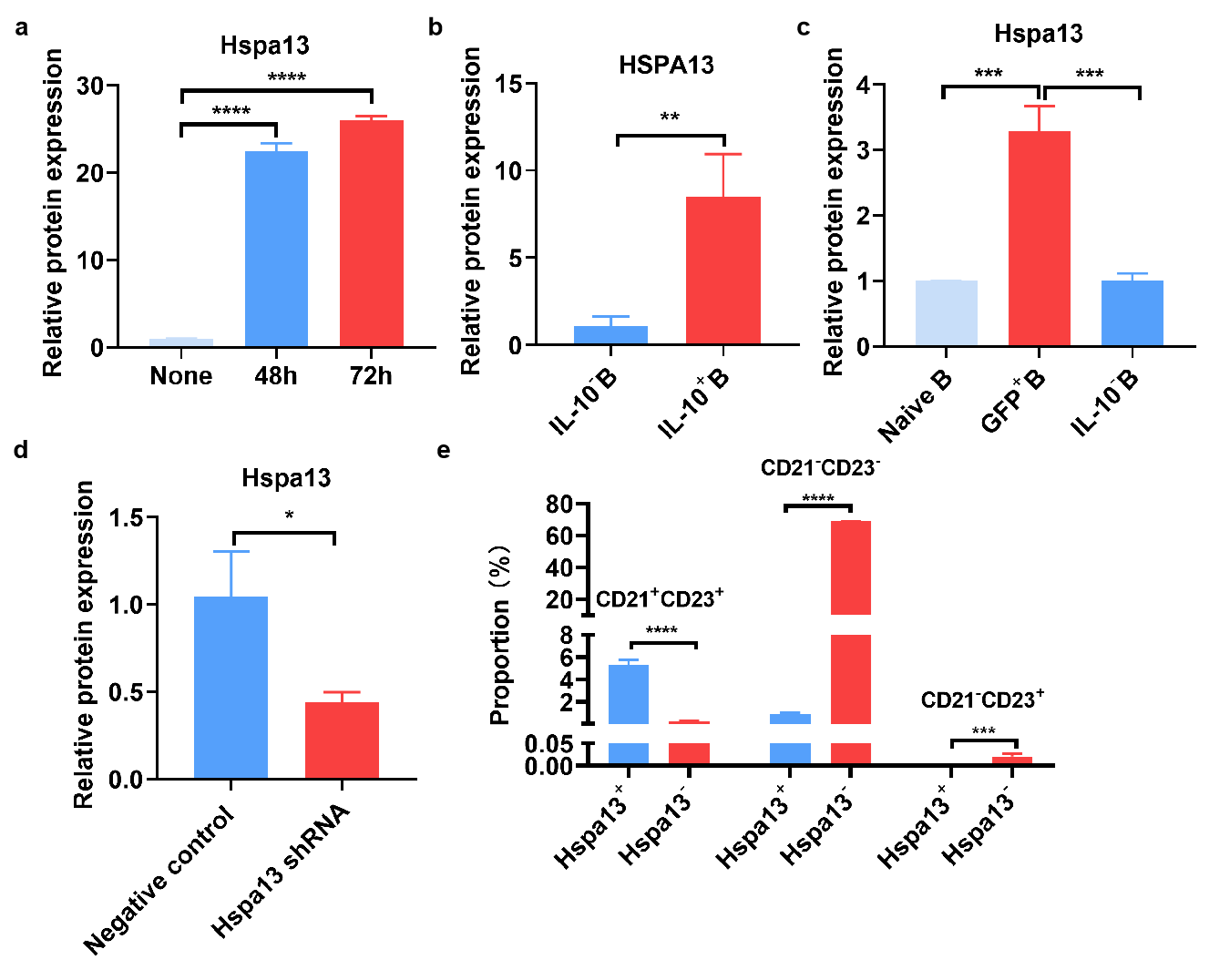


**Figure S1. Statistical analysis of Hspa13 protein expression and Hspa13 positive cell phenotype.** a) Isolated B cells were stimulated (0h, 48h, 72h) for Bregs induction. Protein expression of Hspa13 in stimulated B cells was detected by WB, followed by quantification and statistical analysis. Hspa13 protein expression in stimulated B cells greatly increased (n=3, ****P*<0.001). b) IL-10^+^ B cells were induced, and then purified using a Bregs isolation kit. Protein expressions of Hspa13 in IL-10^+^ B cells and IL-10^-^ B cells were detected by WB, followed by quantification and statistical analysis. Hspa13 protein expression exhibited a significant increase in IL-10^+^ B cells compared to the IL-10^-^ B cells (n=3, ***P*<0.01). c) Naive B cells, GFP^+^ B and GFP^-^ B cells were isolated from IL-10-EGFP reporter tiger mice through FACS. Protein expressions of Hspa13 in naive B cells, GFP^+^ B and GFP^-^ B cells were detected by WB, followed by quantification and statistical analysis. Hspa13 expression in GFP^+^ B cells was much higher than that in naive B cells or GFP- B cells (n=3, ****P*<0.001). d) Hspa13 expression was knockdown in B cells via Hspa13-specific shRNA-expressing lentiviral infection. Protein expression of Hspa13 was detected by WB, followed by quantification and statistical analysis. (n=3, **P*<0.05). e) The percentages of CD21^+^CD23^+,^ CD2^-^CD23^+^, CD21^-^CD23^-^B cells in Hspa13 positive or negative cells were analyzed by FACS, followed by statistical analysis (n=3, ****P*<0.001, *****P*<0.0001). Statistical analyses were performed by two-tailed t-tests between two groups, while two-way ANOVA test were performed between multiple groups.

Figures S2


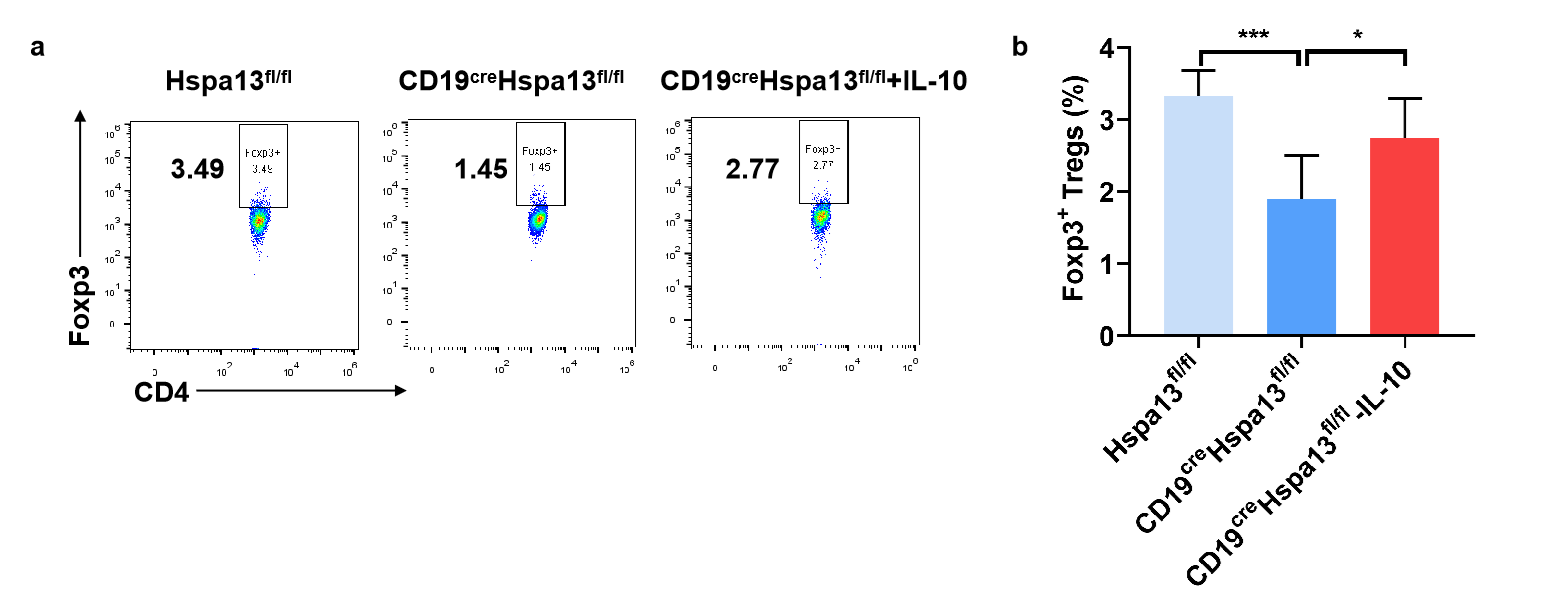


**Figure S2. IL-10 restored Tregs proportion in CD19^cre^Hspa13^fl/fl^.** a) The CD19^cre^Hspa13^fl/fl^ mice we administrated with recombinant IL-10 protein (0.2μg/mouse) by intraperitoneal injection for two times within one week. IL-10 administration restored the decreased Tregs proportion in CD19^cre^Hspa13^fl/fl^ mice by FACS analysis. b) The statistical analysis of Tregs proportion in spleens of IL-10 administrated CD19^cre^Hspa13^fl/fl^ mice (n=6, **P*<0.05, ****P*<0.001).

Figures S3


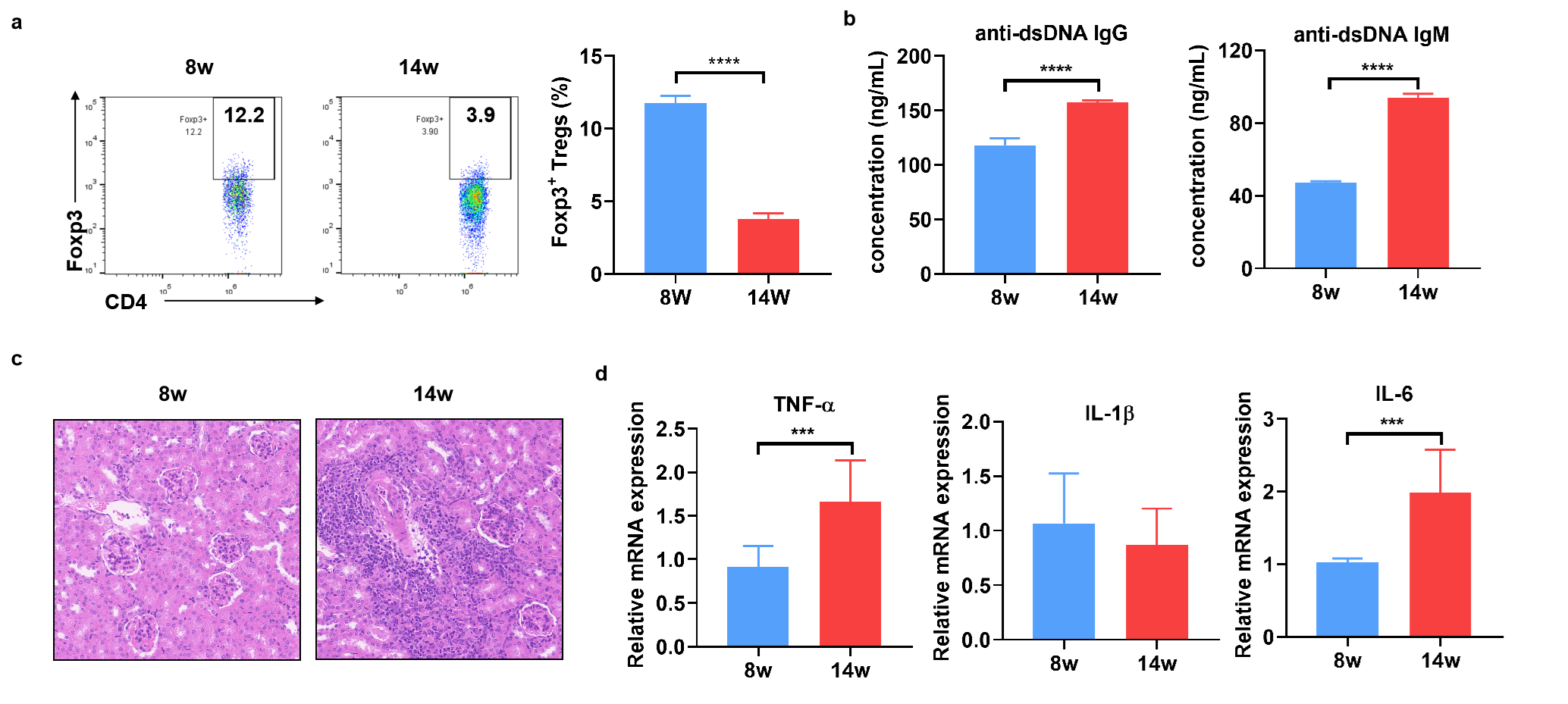


**Figure S3.** **Pathological changes of 14w-MRL/lpr mice.** a) The proportion of Tregs in spleens of 14w-MRL/lpr mice was decreased than that in 8w-MRL/lpr mice by FACS analysis (n=4, *****P*<0.0001). b) The serum anti-dsDNA IgG and IgM antibodies were significantly increased in 14w-MRL/lpr mice compared to that in 8w-MRL/lpr mice by ELISA analysis (n=6, *****P*<0.0001). c) The lupus nephritis especially with severe glomerular pathology and inflammatory cell infiltration in renal of 14w-MRL/lpr mice were observed compared to that in 8w-MRL/lpr mice by HE staining. d) The renal inflammatory factors (IL-1β, IL-6, TNF-α) expressions were significantly increased in 14w-MRL/lpr mice compared to that in 8w-MRL/lpr mice by real-time PCR analysis (n=6, ****P*<0.001). Statistical analyses were performed by two-tailed t-tests between two groups, unless otherwise stated.
